# The Meso- and Bathypelagic Archaeal and Bacterial Communities of the Southern Gulf of Mexico Are Dominated by Nitrifiers and Hydrocarbon Degraders

**DOI:** 10.3390/microorganisms13051106

**Published:** 2025-05-11

**Authors:** Lizt Selene Osorio-Pando, Mario Hernández-Guzmán, Karla Sidón-Ceseña, Yamne Ortega-Saad, Victor F. Camacho-Ibar, Jennyfers Chong-Robles, Asunción Lago-Lestón

**Affiliations:** 1 Posgrado de Ciencias de la Vida, Centro de Investigación Científica y de Educación Superior de Ensenada (CICESE), Ensenada 22860, Baja California, Mexico; lizt@cicese.edu.mx (L.S.O.-P.);; 2 Departamento de Innovación Biomédica, Centro de Investigación Científica y de Educación Superior de Ensenada (CICESE), Ensenada 22860, Baja California, Mexico; hernandezgm@cicese.mx (M.H.-G.); yamnosa@gmail.com (Y.O.-S.); jennyfer.chong@gmail.com (J.C.-R.); 3 Instituto de Investigaciones Oceanológicas, Universidad Autónoma de Baja California (UABC), Ensenada 22860, Baja California, Mexico; vcamacho@uabc.edu.mx

**Keywords:** deep sea, 16S metabarcoding, Gulf of Mexico, microbial community dynamics, ammonia-oxidizing archaea, marine microbes, mesopelagic, bathypelagic

## Abstract

The Gulf of Mexico (GoM) is a complex oceanic basin with a maximum depth of 4000 m. It is a complex hydrodynamic system formed by different water masses with distinctive physical and biological characteristics that shape its rich biodiversity. In this study, as a contribution to better understanding the microbial communities inhabiting the meso- and bathypelagic zones of the Mexican Exclusive Economic Zone (EEZ) of the GoM, an extensive set of seawater samples was collected at three depths (350–3700 m) during three oceanographic cruises. The V4-16S rRNA gene analysis identified Pseudomonadota (27.1 ± 9.8%) and Nitrosopumilales (26.4 ± 2.3%) as the dominant bacterial and archaeal members, respectively. The depth, salinity, and apparent oxygen utilization were key environmental drivers, which explained 35% of the community variability. The mesopelagic zone presented a more homogeneous structure characterized by a nitrifier community, while the bathypelagic was more heterogeneous, with hydrocarbon-degrading bacteria and methanogens serving as the key players. This study is the first to report the archaeal community in the deeper waters of the Mexican EEZ of the GoM, playing crucial roles in the nitrogen and carbon cycles, highlighting the region’s ecological complexity and the need for further research to understand the broader biogeochemical implications of these processes.

## 1. Introduction

The Gulf of Mexico (GoM) is a complex marine ecosystem with unique geological features, diverse biota, and strong ecological interactions. The Gulf is a semi-enclosed basin connected to the Caribbean Sea via the Yucatan Channel and to the Atlantic Ocean through the Florida Strait, facilitating the exchange of water masses that shape its unique hydrography [1,2]. Each water mass has distinctive physical and biological characteristics and is influenced by regional and global ocean currents. The major current influencing the GoM is the Loop Current (LC), which introduces warm and oligotrophic waters from the Caribbean and plays a key role in the circulation patterns of the Gulf.

Oceanic waters are conventionally divided into three primary zones based on the depth: the epipelagic zone (surface layer), the mesopelagic zone (twilight or midwater layer), and the bathypelagic zone (deep sea). This stratification is crucial for understanding marine ecosystems and the distribution of marine organisms, as well as the physical and chemical gradients that characterize each layer. The Gulf water masses are distributed across different surface and subsurface layers. The epipelagic zone (0–200 m depth) hosts several water masses, such as the Caribbean Surface Water (CSW) introduced by the LC and Subtropical Underwater (SUW), whereas the mesopelagic zone (200–1000 m depth) hosts the Tropical Atlantic Central Water (TACW; 300–700 m), which is the location of the relative oxygen minimum zone (ROMZ) and the Antarctic Intermediate Water (AAIW, 700–1000 m). Finally, the bathypelagic zone (1000–4000 m depth) hosts the North Atlantic Deep Water (NADW). Each of these water masses contributes to th vertical stratification and nutrient distribution in the basin [3,4].

The mesopelagic zone is a heterogeneous layer with a strong gradient of oxygen concentration that influences the distribution and activity of the microbial communities, in which ~90% of the organic carbon exported annually from the euphotic zone is respired back into CO_2_ [5,6]. The shallower layers of the GoM are an oligotrophic ecosystem with low net primary production (NPP). However, discrepancies have been reported between the low particulate organic matter (POM) produced in the euphotic zone and the high values exported to the mesopelagic region during the summer, resembling patterns observed in more productive ecosystems [7]. By contrast, the bathypelagic zone is an environment characterized by low temperature (4 °C), high pressure, and stable salinity [8,9]. These deep waters also contain hydrocarbon seeps, brine pools, and occasional hydrothermal activity, creating unique ecological niches for microbial life [10,11], which influences the microbial community structure and function [12].

The assessment of the bacterial and archaeal communities in the water column in large-scale analyses throughout the global ocean has shown that they are structured differently [13,14,15,16,17,18,19]. A set of different variables contributes significantly to these differences. The null or limited autonomous motility of free-living bacterial and archaeal cells, the input of organic material via particulate organic matter through the water column [20], and because differences in density can act as a physical barrier [13,21] are a few of the substantial factors shaping the microbial structure in the open ocean. In the GoM, the information about this is limited and restricted to specific areas, with most studies conducted in the northern region and primarily focused on mesopelagic communities [22], with little representation of the bathypelagic zone [23], or just related to the Deepwater Horizon oil spill [24,25,26].

To our knowledge, there are only two studies describing the bacterial community composition through different water masses in the southern GoM, covering the mesopelagic and bathypelagic zones [27,28]. Both studies described the bacterial structure through the water masses, with little focus on how environmental factors shape the bacterial communities vertically and horizontally. In addition, none provide information about the archaeal community despite their essential function in the Earth’s biogeochemical cycles [29,30]. The current knowledge about the paramount importance of the archaeal in the C and N cycles reinforces our need to overcome the lack of information about their contribution in the deep waters of marine environments such as those in the GoM. Similar to bacterial assemblages, the previous research on this community across the water column has shown a geographical limitation restricted to the United States Exclusive Economic Zone (EEZ) of the GoM [22,23,31,32] or focused on extreme environments such as the hypoxic zone [32], gas hydrate systems [33], and cold-asphalt volcanoes [34]. For the EEZ of Mexico, only a study from the Yucatan platform (ca. 180 m depth) has been performed [35]. However, there is currently a lack of information about the meso- and bathypelagic layers of the southern GoM, particularly regarding the role of these communities in deep-water ecosystems and their influence on global cycles.

This study aims to address this gap by expanding our understanding of the microbial communities that inhabit the meso- and bathypelagic zones of the southern GoM, focusing on (i) their spatial distribution, (ii) temporal dynamics, and (iii) putative ecological roles. In doing so, we not only expand the number of sampled locations compared to previous studies but also place our findings within the broader environmental context of the region. Furthermore, our study describes for the first time the archaeal communities in the deep waters of the southern GoM, where their role has been largely overlooked. This contribution is crucial for improving our understanding of microbial diversity and ecological processes in the deep ocean, which is essential for predicting the impact of environmental changes on these poorly explored ecosystems.

## 2. Materials and Methods

### 2.1. Sample Collection and Sample Characterization

**Figure 1:**
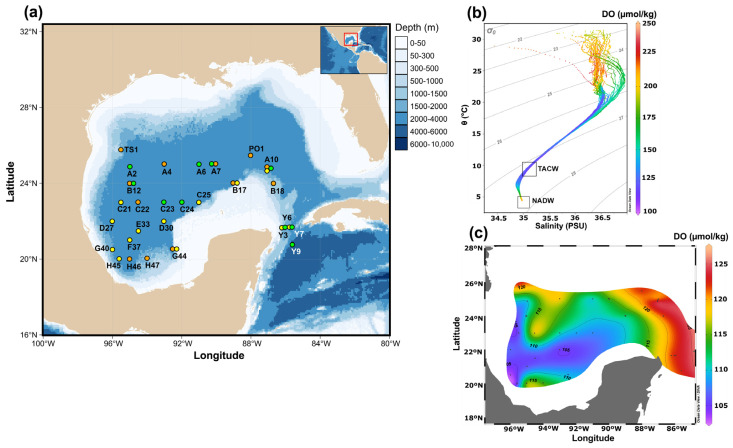
The Gulf of Mexico. (**a**) Map of the study area showing the seawater sampling locations, with the colors indicating the oceanographic campaigns (XIXIMI-05: orange; XIXIMI-06: yellow; XIXIMI-07: green). (**b**) Temperature–salinity (T-S) diagram and dissolved oxygen (DO, µmol kg^−1^) from the data obtained with the SBE 911plus CTD (see Section 2.1). The Tropical Atlantic Central Water core (TACW, low DO) and the North Atlantic Deep Water (NADW, high DO) are indicated with empty squares in the T-S diagram. (**c**) DO (µmol kg^−1^) distribution in the isopycnal 27.1 kg m^−3^ (TACW core) from all the samples.

Ninety-five seawater samples were collected from 35 stations in the mesopelagic and bathypelagic zones within the Mexican Exclusive Economic Zone (EEZ) of the GoM. Sampling was performed aboard the R/V *Justo Sierra* (UNAM) during three oceanographic campaigns: XIXIMI-05 (10–25 June 2016; n = 12), XIXIMI-06 (5 August to 8 September 2017; n = 13), and XIXIMI-07 (9 May to 2 June 2019; n = 10) (Figure 1). The stations were categorized as either “shallow” or “deep” based on the maximum sampling depth relative to 1000 m. Shallow stations reached depths of up to 1000 m, while deep stations extended close to the seafloor. Samples were collected mostly from three distinct depths at each station, with a few exceptions due to logistical issues. For the shallow stations, samples were taken at (i) the relative oxygen minimum zone (ROMZ, 350–600 m, oxygen concentration was lower compared to surrounding waters but not suboxic or hypoxic), (ii) 800 m, and (iii) 1000 m. For the deep stations, samples were taken at (i) the ROMZ, (ii) 1000 m, and iii) the bottom (BTM, 1100–3700 m, ca. 30 m above the seafloor). A detailed description of the stations sampled is given in Table S1.

Seawater was collected using 20 L Niskin bottles. At each station and depth, 6 L of seawater was taken and pre-filtered directly from the mouth of the Niskin bottles using a 200 μm Nitex mesh. The pre-filtered seawater was then divided into two 3 L samples and filtered through different pore sizes via an in-line vacuum filtration system connected to an Eyela A-1000-S pump. The system included an initial 0.8 μm pore size of 47 mm track-etched polycarbonate membrane (Whatman^®^, Nuclepore™, Maidstone, UK), followed by a 0.2 μm Sterivex™ filter (Merck Millipore, Burlington, MA, USA). The filters were immediately frozen in liquid nitrogen onboard and stored at −80 °C at the laboratory until further DNA extraction. For this study, only the fraction collected through the Sterivex filters (0.2–0.8 μm), which contained free-living prokaryotes, was further processed and analyzed.

During sampling, physical and chemical characteristics, such as the depth (m), temperature (°C), salinity (practical salinity units: PSUs), and dissolved oxygen (DO), were measured using an SBE 911plus CTD (Sea-Bird Scientific, Bellevue, WA, USA). The nitrate (NO_3_^−^ + NO_2_^−^) and phosphate (PO_4_^3−^) concentrations were determined following the procedure described by Linacre et al. [36]. The apparent oxygen utilization (AOU) was calculated as the difference between the observed oxygen concentration and the saturation level. The saturated DO was determined using the measured temperature and salinity, as described by Garcia and Gordon [37]. The conservative temperature and absolute salinity were determined using the TEOS-10 equations [38].

### 2.2. Total DNA Extraction, V4-16S rRNA Amplification and Sequencing

The total DNA was extracted from the Sterivex™ filters collected per sample using a modified phenol–chloroform method to increase the yield of high-molecular-weight DNA [39]. After the DNA extraction, both sample replicas were combined and used for the subsequent PCR amplification in triplicate. Amplicon libraries were prepared via one-step PCR using the dual-indexing strategy described by Kozich et al. [40]. Briefly, the hypervariable V4 region of the 16S rRNA gene was amplified using the 515F and 806R primers designed by Caporaso et al. for bacterial and archaeal identification [41]. In addition to the taxonomic marker, the primers contained a 2 nt linker, a 10 nt pad sequence, an 8 bp index (unique per-sample), and the Illumina adapter [40]. Each DNA sample was amplified in triplicate. The PCR reactions (20 μL) contained 10 ng DNA, 0.4 μM of each primer, 0.625 U MyTaq™ DNA polymerase (Bioline^®^, Memphis, TN, USA), and 1×MyTaq™ reaction buffer. The thermal conditions for amplification were as follows: 95 °C for 2 min, followed by 30 cycles of 95 °C for 20 s, 55 °C for 15 s, 72 °C for 5 min, with a final extension at 72 °C for 7 min. A negative PCR reaction (without template) was included during the amplification. The PCR triplicates were pooled, quantified, and then purified and normalized using a SequalPrep™ Normalization Plate Kit, 96-well (Applied Biosystems™, Thermo Fisher Scientific^®^, Waltham, MA, USA). Quantification was performed using a Qubit 3.0 Fluorometer and the dsDNA HS Assay Kit (Invitrogen™, Thermo Fisher Scientific^®^). Sequencing was performed using the MiSeq Reagent Kit v2 (300-cycles) including 10% PhiX (Illumina©) and performed in the Illumina© MiSeq sequencer at the CICESE facilities.

The raw sequences are publicly available in the Sequence Read Archive (SRA) database under the NCBI’s BioProject accession number PRJNA1141103.

### 2.3. Sequencing Data Analyses

The demultiplexed sequences underwent an initial quality assessment with the FastQC tool v0.12 [42] Sequences with a Q-Score ≥ 20 were retained for the downstream analyses. The raw sequences were processed using the DADA2 package v1.16 for the amplicon sequence variants (ASVs) inference and taxonomic annotation [43] within the R environment v4.2 [44]. The DADA2 pipeline v1.8, available at https://benjjneb.github.io/dada2/tutorial_1_8.html (accessed on 10 February 2024), was followed with the default settings, except for the trimming parameters, which were set to 149 bp and 140 bp for the forward and reverse reads, respectively. Taxonomy annotation was performed using the DADA2 pre-formatted reference database based on SILVA v.138 [45], and through a naive implementation of the Bayesian classifier. Subsequently, a neighbor-joining rooted phylogenetic tree was constructed by employing the msa v.1.22 [46], phangorn v.2.6.2 [47], and ape v.5.4-1 [48] packages in the R environment. The taxonomic information, representative sequences, frequencies and phylogenetic relationships were later converted to a phyloseq object in the R environment through the phyloseq package v1.32 [49] for summarizing, taxonomic parsing and collapsing. The mitochondrial, chloroplast, and ASVs containing < 2 reads were excluded before the downstream analyses.

The microbial gene content and putative functional information of the bacterial and archaeal communities was inferred using PICRUSt2 (Phylogenetic Investigation of Communities by Reconstruction of Unobserved States) through the full pipeline script with the default settings (available at https://github.com/picrust/picrust2/wiki/Full-pipeline-script (accessed on 4 April 2024)) [50]. The reliability of the predictions was validated using the “Nearest sequenced taxon index” values (NSTI ≤ 2).

### 2.4. Ecological and Statistical Analyses

The alpha-diversity metrics, including the species richness (here ASVs), Shannon, Simpson, and Gini indices, were determined using a rarefied dataset for the sample with the lowest number of observations (i.e., 39,028) via the “alpha” function of the microbiome v.1.12 package [51]. Before the statistical comparison, the diversity datasets were normalized with the “transformTukey” function from the rcompanion v.2.4.1 package [52]. A one-way ANOVA followed by a Tukey’s HSD test were used to determine the differences in the alpha-diversity metrics using the depth, region and oceanographic campaigns as fixed factors (Table S1). The Gini index, a coefficient used to assess the specific degree of evenness, was determined based on the relative abundance of the ASVs for each depth [13].

The beta diversity was assessed using non-metric multidimensional scaling (NMDS) based on both the weighted and unweighted UniFrac distances [53], as determined on the rarefied dataset (as described above). Differences in the microbial community structure were determined using a permutational analysis of variance (PERMANOVA) with the “adonis2” function, based on the UniFrac distance matrices and 9999 permutations, using the vegan v2.6 package [54]. The depth, region and oceanographic campaigns were included as fixed factors in the PERMANOVA analyses. Following a statistically significant result from the PERMANOVA, post hoc pairwise comparisons among the categories were conducted using the “pairwise.adonis” function [55]. The homogeneity of variance test for multivariate analyses was conducted (“betadisper”) before each PERMANOVA analysis.

To identify key environmental variables influencing the most variability within the archaeal and bacterial communities, a distance-based redundancy analysis (db-RDA) was performed using the Euclidean distance of the clr-transformed data (“rda” function) and the standardized environmental variables (using the “scale” function), both via the vegan package. The statistical significance of the db-RDA model was evaluated, and the contribution of each environmental variable to the variation in community composition was assessed using an ANOVA-like permutation test [56]. A variance inflation factor (VIF) test was employed, ensuring the VIF values remained < 5 to avoid collinearity. The db-RDA analysis was conducted separately on distinct datasets as follows: (i) ASVs corresponding to the entire community, (ii) ASVs from the dominant biosphere (DB) (ASVs representing ≥ 1% within a sample and with a mean relative abundance of ≥0.1% per depth layer), and (iii) ASVs from the rare biosphere (RB) (ASVs representing ≤ 0.01% within a sample and with a mean relative abundance of ≤ 0.001% per depth layer). Classification thresholds for defining dominant or rare ASVs were used to avoid any overlap between both [17]. Intersection analysis using the UpSet plots (available via the Shiny app at https://upset.app/ (accessed on 7 August 2024)) [57] was employed to visualize the shared and unique ASVs among the depths, utilizing datasets for the entire community or for the DB or RB, as appropriate.

The non-parametric Kruskal–Wallis test, via the “aldex.kw” and “aldex.effect” functions of the ALDEx2 package v1.28 [58,59,60], was used to determine the differences in the microbial groups’ abundance among the different depths in a pairwise manner (differential abundance analyses, DA) and applied using the clr-transformed datasets. The effect size metric was used to select those groups displaying significant differential abundance based on a cutoff ≥ 1 (which means a “large effect size”) following the developer’s recommendations. The DA analysis focused on those ASVs with a prevalence of at least 10% of the samples when two depths were compared.

## 3. Results

### 3.1. Environmental Context

The physical and chemical characteristics of the seawater exhibited large and significantly variation among the studied depths (Table 1 and Table S2). Samples from the ROMZ were collected from the TACW core water mass, which was characterized by a conservative temperature of (mean ± sd) 10.1 ± 0.7 °C and an absolute salinity of 35.41 ± 0.10 g kg^−^¹, falling within the isopycnal range of 27–27.2 kg m^−3^. As expected, the DO concentrations were low, with greater values than 120 µmol kg^−1^ at the stations located in the LC and its detached anticyclonic eddies (i.e., PO1, A10, B18, Y6, Y7 and Y9), and lower values than 110 µmol kg^−1^ at those stations closer to the coast (H45, G40, G44, C21). The higher values of the AOU were found in the ROMZ (166.8 ± 4 μmol kg^−1^), while the lowest were observed at the BTM (116.8 ± 4 μmol kg^−1^). The AOU distribution exhibited a spatial pattern opposite to that of the DO, with higher values at stations near the coast and decreasing at stations within the LC.

All the samples collected at 800 m came from the AAIW water mass (27.4–27.5 kg m^−3^ isopycnals), while those at a 1000 m depth were lying in the AAIW or at the transition between the AAIW and the NADW (27.6 kg m^−3^) (Tables S1 and S2). At these depths, the DO tended to increase and the AOU tended to decrease up to the BTM. Finally, the BTM was in the NADW water mass, with a conservative temperature of 4.1 ± 0.1, absolute salinity of 35.15 ± 0.00, and isopycnal of 27.7 kg m^−3^. The DO concentration at the BTM was approximately twice the DO measured at the ROMZ. The nitrate (NO_3_^−^ + NO_2_^−^) and phosphate (PO₄³^−^) concentrations were higher at 800 m compared to the other depths, with the BTM samples showing the lowest values (Table 1).

### 3.2. Structure of Bacterial and Archaeal Communities in the Aphotic Zone of the GoM

A total of 9,502,554 high-quality and filtered sequences were generated in this study. The sequences were grouped into 8237 ASVs, out of which 7401 (~90%) were identified as bacteria and 836 (10%) as archaea. The bacterial community comprised 40 phyla, 88 classes, 206 orders, 234 families and 378 genera, whereas the archaeal community comprised 7 phyla, 7 classes, 12 orders, 11 families and 7 genera. Taxonomic annotation of the ASVs decreased with each taxonomic rank; nearly 97% of bacterial ASVs were assigned up to the phylum, 80.3% up to the class, 70.2% up to the order, and 26.9% at the genus level. Similarly, more than 98% of the archaeal ASVs were assigned up to the phylum, class and order, but less than 5% up to the genus rank. Regarding the distribution among depths, the largest number of ASVs was found at 1000 m (number of ASVs: 4276), followed by the ROMZ (4046), BTM (3043) and 800 m (2823). The rarefaction curves approached the plateau, indicating enough sequencing effort to capture most of the microbial diversity (Figure S1a).

**Figure 2:**
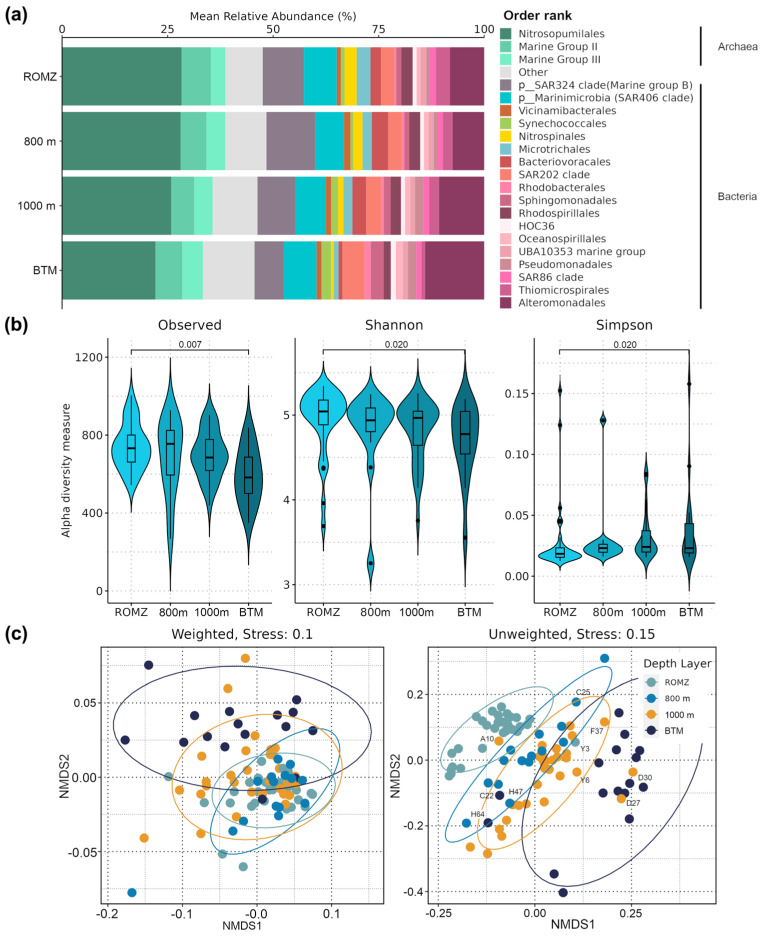
Bacterial and archaeal community structure across depths in waters of the Gulf of Mexico. (**a**) Bar plot showing the average relative abundances (%) of the bacterial and archaeal community up to the order level. Low abundance groups (<1% relative abundance) were grouped as “Other”. (**b**) Violin plot showing the alpha diversity of the prokaryotic community using traditional indices: species richness (“Observed”; here ASVs), Shannon and Simpson indices. The *p*-values indicate significant differences between depths, i.e., pairwise comparison (Tukey’s HSD test, *p* < 0.05). (**c**) Non-metric multidimensional scaling (NMDS) ordination plots based on the weighted and unweighted UniFrac distances; labeled stations represent those that overlap two different depths.

The overall community comprised 62.5 ± 6.9% and 37.5 ± 6.9% (mean ± sd of the relative abundances of all the samples) of bacterial and archaeal phylotypes, respectively. Proteobacteria (currently Pseudomonadota) (27.1 ± 9.8%) dominated the bacterial community, followed by the two archaeal phyla Thermoproteota (26.5 ± 5.6%) and Thermoplasmatota (10.4 ± 2.6%). At the order level, nineteen groups exhibited a relative abundance > 1% (Figure 2a). Nitrosopumilales (26.4 ± 2.3%) was the dominant group across depths, followed by Alteromonas (9.6 ± 2.3%), Marine Group (MG) II and MG-III (6.2% and 4.3%, respectively), SAR202 (3.8%), Bacteriovoracales (2.7%), Thiomicrospirales (2.7%), Rhodospirillales (2.3%), Microtrichales (2.2%), Nitrospinales (2.1%), Synechococcales (1.8%), Sphingomonadales (1.7%), Pseudomonadales, SAR86 and UBA10353 (1.7%, 1.4%, and 1.2%, respectively), and Oceanospirillales, Vicinamibacterales and HOC36 (1.5%, 1.4%, and 1.2%, respectively). Unknown phylotypes of the phyla “SAR324 clade (Marine Group B)” and “Marinimicrobia (SAR406 clade)” were also among the most abundant, with 9.6% and 7.4% relative abundance across depths, respectively.

The relative abundance of various bacterial and archaeal groups showed a contrasting response with an increasing depth. For instance, the relative abundance of dominant groups such as Nitrosopumilales, Bacteriovoracales, Thiomicrospirales, Rhodospirillales, Microtrichales, Nitrospinales and SAR86 decreased with depth, whilst Alteromonas, Pseudomonadales, Oceanospiralles, Sphingomonadales, Rhodobacterales, SAR202, and Synechococcales showed the opposite pattern. Members of MG-II, MG-III, HOC36, UBA10353, and Vicinamibacterales remained relatively similar across depths (Figure 2a).

Regarding the alpha diversity, the results showed that the species richness (here ASVs) and diversity based on the Shannon index significantly decreased with depth, whereas the Simpson index showed the opposite (ANOVA: F ≤ 4.78, *p* ≤ 0.039; Figure 2b). Differences in richness and diversity were identified between the ROMZ and the BTM (Tukey’s HSD, *p* ≤ 0.020; Figure 2b). In addition, the Gini index showed that most of the ASVs presented an uneven distribution between depths; the BTM showed the most uneven community (92.3% of ASVs with Gini ≥ 0.5), whereas 800 m presented the most even (87.7% of ASVs with Gini ≥ 0.5) (Figure S1b). The richness and diversity showed no significant variations between sampling regions (Figure S2a,b); however, the diversity varied significantly between oceanographic campaigns (i.e., temporal variability) (*p* ≤ 0.05; Figure S2c,d).

The beta-diversity analyses showed significant differences in the microbial structure assembly related to the depth and sampling region (Figure 2 and Figure S2, Table 2). The NMDS ordination clearly separated the microbial community by depth, irrespective of the UniFrac distances (Figure 2c), with major differences observed between the mesopelagic (i.e., samples collected at the ROMZ, 800 m, and 1000 m) and bathypelagic zones. The differences within the mesopelagic zone were smaller (Figure 2c). However, a higher degree of sample clustering among the depths was observed when the abundance of phylotypes was not considered, i.e., unweighted UniFrac (Figure 2c), thus separating the community more strongly. The PERMANOVA analysis demonstrated that the differences observed in the prokaryotic community structure among the depths were significant (Figure 2C and Table 2). Moreover, a detailed between-depths analysis using pairwise comparisons (i.e., post hoc PERMANOVA) revealed that differences were primarily observed between the ROMZ, 1000 m, and BTM (Table S3, R^2^ ≤ 18%, *p* ≤ 0.006).

Furthermore, the PERMANOVA indicated that the community assembly presented significantly differences among regions (R^2^ ≤ 9.6%, *p* ≤ 0.003). Ordination analyses showed that samples from the south presented a more homogeneous microbial composition compared to all the other regions, but only when the phylotype abundances were considered (see weighted UniFrac; Figure S2a,b). Moreover, pairwise comparisons between regions showed that the major differences in the prokaryotic assembly were between the LC and both the center and south regions and mainly in the ROMZ and, in some cases, down to 1000 m (Figure S2a,b, Table S3).

Finally, given the significant differences in the dispersion of samples between oceanographic campaigns, as shown by the betadisper results (Figure S2b,c, *p* ≤ 0.041), the effect of temporal variability on the prokaryotic community structure could not be confirmed.

### 3.3. Effect of Environment on the Bacterial and Archaeal Community Structure

**Figure 3:**
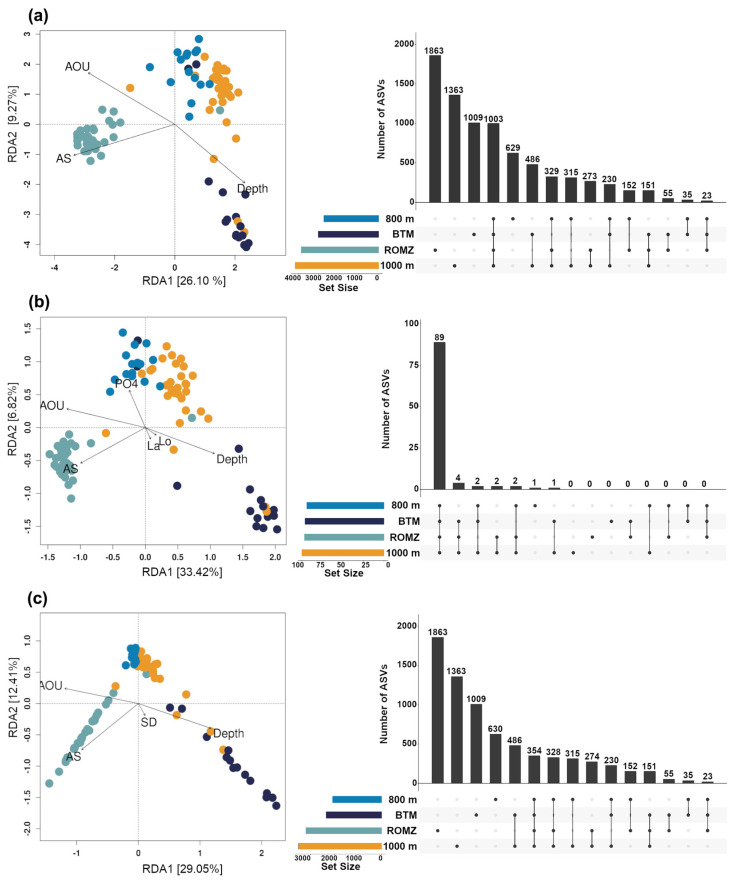
Distance-based redundancy analysis (db-RDA) of the bacterial and archaeal communities constrained by seawater physicochemical characteristics. The db-RDA analysis as determined on (**a**) the entire prokaryotic community (all ASVs); (**b**) the dominant biosphere (ASVs representing ≥ 1% per sample and with a mean relative abundance ≥0.1% per depth layer); and (**c**) the rare biosphere (ASVs representing ≤0.01% per sample and with a mean relative abundance of ≤0.001% per depth layer). Upset plots displayed on the right-hand side of each db-RDA show the number of unique (single and black-filled dots below bars) and shared (multiple and line-connected black dots below bars) ASVs between depths (relative oxygen minimum zone (ROMZ), 800 m, 1000 m and BTM).

The depth, AS and AOU collectively explained 35% of the variability for the entire community (Figure 3a and Table S4, *p* ≤ 0.005). The bacterial and archaeal structures in the ROMZ exhibited a strong and positive correlation with the AS, whereas 800 m and 1000 m showed a negative one. Moreover, the BTM displayed a positive correlation with the depth and negative with the AOU. The correlation of the aforementioned variables with the dominant (DB) and rare biosphere (RB) was relatively similar to those observed with the entire community (Figure 3b,c); however, some differences were also noted. For example, the DB at 800 m was positively correlated with the phosphate content, whereas the RB at the BTM was positively correlated with the seafloor depth. Overall, environmental variables explained 47% and 44% of the prokaryotic variability for the DB and RB, respectively (Figure 3b,c).

When considering the entire prokaryotic community, intersection analyses showed that 42% of all the phylotypes (3052 ASVs) were shared for either two or more depths, but only 13.8% (1003 ASVs) were identified in all four depths (i.e., core phylotypes) (Figure 3a). The analyses also showed that the ROMZ presented the greatest number of unique ASVs (25.6%), followed by 1000 m (18.8%) and the BTM (13.9%). Furthermore, a total of 101 ASVs were identified as the dominant biosphere (DB), out of which 81% were shared between the four depths (i.e., the core phylotypes) (Figure 3b). On the other hand, the number of ASVs shared among all the depth layers in the RB dataset was lower than expected compared to the result using the entire community, due to the ASVs not classified in either the DB or RB datasets, 649 (8.2%). Finally, similarly to the entire community in the RB dataset, the depth layer pairs that shared the most ASVs were 800 m–1000 m and 1000 m–BTM (17%), and the least, ROMZ–BTM (8%).

### 3.4. Analysis of the Prokaryotic Community in the Bathypelagic Zone

**Figure 4:**
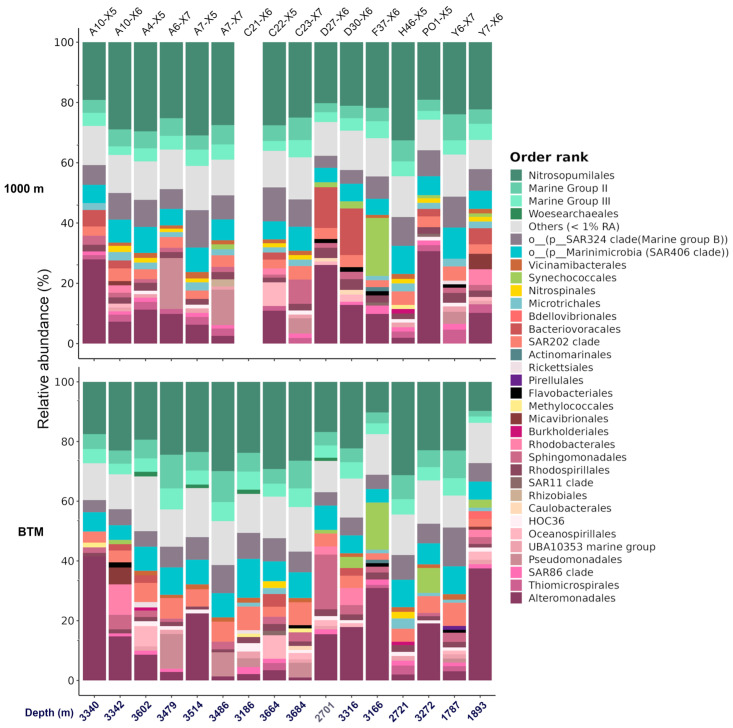
Comparison of the relative abundance of the bacterial and archaeal community between 1000 m and the BTM in the Gulf of Mexico. Bar plot showing the relative abundance (%) of samples from 1000 m and the BTM. Numbers displayed on the bottom x-axis represent the depth (meters below sea level) at which the sample was taken (see Table S2 for details), whereas labels on the upper x-axis display the station name.

A large variability in the microbial community structure was observed in the samples from the bathypelagic zone (Figure 2c and Figure S1b), in contrast to the relatively small variation in their physicochemical characteristics (Table 1). Distinct patterns of abundance were observed across the different stations at the BTM. Among the most abundant orders, we found Nitrosopumilales (described with more detail in the following section) (Figure 2a and Figure 4), and Alteromonadales, a well-known order containing phylotypes with hydrocarbon degradation capabilities, was the second most dominant group (9.6% relative abundance of all the samples), mainly due to the presence of the genus *Alteromonas* (representing ca. 90% of this group) in A10-X5, D27-X6, PO1-X5, A7-X5, and Y7-X6 (Figure 4), while in D30-X6 and F37-X6, *Alteromonas* and *Psychromonas* were the dominant genus of Alteromonadales.

Other groups also related to the degradation of hydrocarbons exhibited high relative abundances at specific stations. For example, members of Pseudomonadales (genus *Acinetobacter*) were the more abundant at stations C23-X7, A6-X7, and A7-X7, Oceanospirillales (genus *Alcanivorax*) at C22-X5 and A4-X5, Sphingomonadales (genus *Erythrobacter*) at D27-X6 and A10-X6, and Rhodobacterales (genera *Limimaricola* and *Pelagibaca*) at A10-X6 and D30-X6 (Figure 4). Six stations out of the fourteen in the BTM that exhibited high abundance of microorganisms involved in hydrocarbon degradation displayed the same pattern at 1000 m as the BTM, i.e., A10-X5, D27-X6, PO1-X5, A6-X7, A7-X7 y C22-X5.

Noticeably, *Prochlorococcus* MIT9313 (order Synechococcales) showed a high relative abundance at stations PO1-X5, D30-X6 and F37-X6, whereas the genus *Micavibrionaceae* (order Micavibrionales) was highly abundant in sample A10-X6 station (Figure 4).

The post hoc PERMANOVA analyses indicated significantly differences between the BTM and 1000 m only when the microbial abundance was not considered (i.e., unweighted UniFrac; Figure 2c and Table S3). This suggests that the major differences between the BTM and 1000 m were mainly due to the low-abundance phylotypes. This was reinforced by the fact that a large number of significantly and differentially abundant phylotypes were those with low abundance (Figure S3). Bacterial phylotypes within orders such as SAR202, Rhodospirillales (mainly Magnetospiraceae), Methylococcales, and Planctomycetales, and those within the candidate phylum PAUC34f, were significant and differentially more abundant at the BTM. In contrast, phylotypes from Nitrospinales (mainly from the genus *Nitrospina*), Microtrichales and the SAR324 clade (mainly within Marine Group B) were more abundant at 1000 m (Figure S3).

### 3.5. Diversity and Distribution of Archaea in the Southern GoM

To our knowledge, there is still a lack of information concerning the archaeal community composition and diversity in the southern GoM. As such, we describe this paramount community in more detail and independent from that of bacteria.

**Figure 5:**
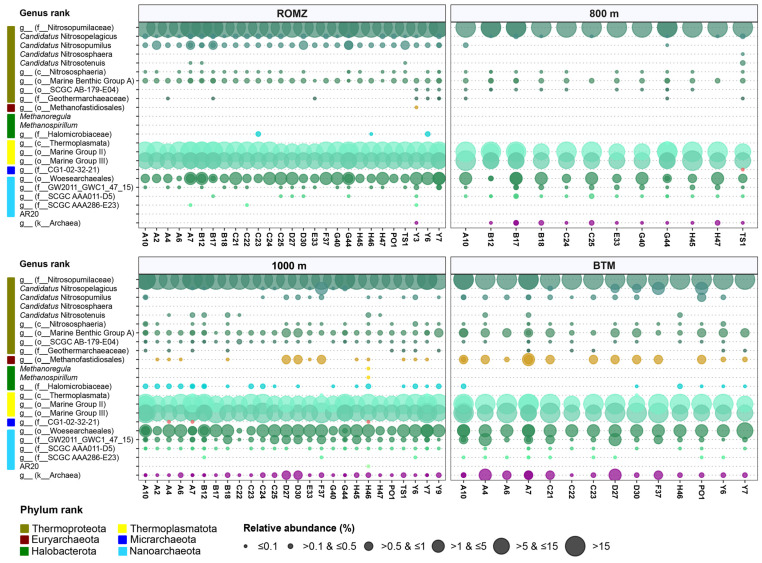
Bubble plot of the archaeal community’s mean relative abundance at the genus level and their distribution pattern among the depths in the Gulf of Mexico. Bubble size represents the categorical values of the relative abundance (%) of groups on a per sample basis.

The overall archaeal community comprised six phyla, with Thermoproteota (solely the Nitrososphaeria class (formerly Thaumarchaeota phylum): 70.4 ± 5.5% relative abundance across all the samples), Thermoplasmatota (solely Thermoplasmata: 27.69 ± 4.97%) and Nanoarchaeota (solely Nanoarchaeia: 1.5 ± 0.9%) dominating the community (Figure S4a,b). The community comprised twelve groups up to the order level, with Nitrosopumilales dominating (70.1 ± 5.6%), followed by MG-II (16.4 ± 3.4%), MG-III (11.3 ± 3.2%) and Woesearchaeales (Figure S4b). Together, these groups accounted for approximately ≥ 95% of the relative abundance of all the phylotypes (Figure 5 and Figure S4a,b).

Up to the genus level, the community was dominated by unknown phylotypes of Nitrosopumilaceae (69.8 ± 5.7%), followed by unknown phylotypes from the orders MG-II (16.4 ± 3.4) and MG-III (11.3 ± 3.2%) (Figure 5). Only four candidate genera were identified, with *C.* Nitrosopumilus (0.2 ± 0.2%) and *C*. Nitrosopelagicus (0.1 ± 0.3%) being the most abundant among them. Additionally, a minor abundance of members within *Methanospirillum* and *Methanoregula* was also identified (≤0.01%) (Figure 5).

The archaeal richness and diversity were significantly affected by the depth (Figure S4c). The richness was significantly greater at the ROMZ compared to the BTM (*p* ≤ 0.001). The diversity, on the other hand, showed an interesting pattern: a similar Shannon diversity was observed through the depths, whilst a significantly greater Simpson diversity was observed at the BTM compared to all the more surficial waters (*p* ≤ 0.001, Figure S4c).

The dominance of the archaeal phylotypes varied with the depth. For instance, members of Nitrosopumilales decreased in the following order: ROMZ (28.3% average relative abundance across depths) > 800 m (28.1%) > 1000 m (25.9%) > BTM (21.1%). A similar trend was observed for members within MG-II. Contrastingly, members within MG-III, Woesearchaeales and Methanofastidiosales showed the opposite (Figure 5 and Figure S4a,b). Genera such as *C*. Nitrosopelagicus and *C*. Nitrosopumilus were identified mostly in the ROMZ and BTM, whereas *C.* Nitrosotenuis and unknown phylotypes of both Methanofastidiosales and Halomicrobiaceae were mostly found at 1000 m and the BTM. Moreover, unknown phylotypes of different families within Woesearchaeales presented higher relative abundance at lower depths (1000 m and BTM) (Figure 5). It is worth noticing that the unidentified archaeal phylotypes increased with the depth (0.1–5% relative abundance per sample).

The analysis performed for the identification of the core community showed the preference of a reduced number of phylotypes for a defined depth. The core archaeal community comprised 163 out of the 836 identified ASVs, and interestingly, the samples taken at the ROMZ showed a relatively similar number of unique phylotypes (Figure S3c). Grouped at the order level, phylotypes unique to both the ROMZ and BTM belonged mainly to MG-III and Nitrosopumilales, whereas those unique to both 800 m and 1000 m belonged mainly to Nitrosopumilales and MG-III. This might suggest a preference of specific phylotypes for different depths.

Finally, major differences in the archaeal community were observed when comparing the ROMZ against the BTM based on the number of differentially abundant phylotypes (Figure S5). Moreover, beta-diversity analyses showed that NMDS similarly clustered the community by depth, with the PERMANOVA indicating significantly variations irrespective of the metric used (Figure S3c, *p* ≤ 0.001, R^2^ ≤ 43%).

### 3.6. Metabolic Potential of Bacterial and Archaeal Communities Across Water Column Depths of the GoM

Differential abundance analysis showed that a reduced number of metabolic pathways were significantly affected by the depth (*p* ≤ 0.001; Table S5). Most of these pathways were differentially abundant, primarily between the ROMZ layer and the two deeper layers, i.e., 1000 m and BTM. Samples taken at the ROMZ exhibited a greater abundance of genes associated with osmoprotection (glycine betaine degradation I and S-adenosyl-L-methionine cycle I) and active cellular metabolism (superpathway of sulfolactate degradation) compared to 1000 m. The methanogenesis pathway was significantly greater in the BTM layer compared to the ROMZ, whilst the latter showed a greater abundance of genes related to the degradation of aromatic compounds (catechol degradation to 2-oxopent-4-enoate II, catechol degradation II (meta-cleavage pathway), 2-aminophenol degradation), amino acid metabolism (L-tryptophan degradation IX, L-tryptophan degradation XII (*Geobacillus*), L-tryptophan degradation to 2-amino-3-carboxymuconate semialdehyde, L-valine degradation I), nitrogen cycling (assimilatory nitrate reduction VI), and the synthesis of essential cofactors (superpathway of thiamin diphosphate biosynthesis I, superpathway of thiamin diphosphate biosynthesis II, thiazole biosynthesis I (*E*. *coli*), thiazole biosynthesis II (*Bacillus*), β-alanine biosynthesis II).

## 4. Discussion

In this study, we assessed the bacterial and archaeal communities from the deep-water region of the southern Gulf of Mexico (GoM) as a contribution to the knowledge of free-living microorganisms’ ecology and their function in the dark and deep ocean (below 200 m depth). We provide the more complete set of data for the southern GoM up to date, including the first description of the archaeal community for the mesopelagic (200–1000 m depth) and bathypelagic zones (1000–4000 m depth) to contribute to the knowledge of the microbial communities of these understudied zones.

### 4.1. Horizontal Distribution of the Microbial Communities in the Mesopelagic Zone Is Shaped by the Oxygen Utilization

Our study showed that the whole microbial community structure was shaped by the depth, AS and AOU. The prokaryote richness and diversity were higher at stations located in the more superficial waters in the mesopelagic zone. This pattern contrasts with findings from the oxygen minimum layer (DO < 100 µmol kg^−1^) of highly productive Atlantic waters, where a decrease in diversity and richness was observed in the mesopelagic layer compared with higher values in the bathypelagic zone [18,61]. However, this study area of the GoM exhibited relatively high DO values (>100 µmol kg^−1^) because it is not a real oxygen minimum zone (OMZ); the oxygen concentrations in the ROMZ were just lower than at surrounding depths and they do not reach the extreme hypoxic conditions observed in other OMZs.

The AOU was a significant factor shaping the microbial community structure, particularly in the ROMZ, where substantial differences were observed between the southern GoM and the LC region. This variation was likely driven by the high productivity levels in the epipelagic zone at the stations influenced by the semi-permanent cyclonic eddy in the southern region and those located near to the coast (stations H47-X5, H46-X5, G44-X5, TS-X5). These conditions enhanced the carbon export and higher respiration rates in the mesopelagic zone [62]. In contrast, the LC can even help oxygenate the ROMZ, enriching this layer inside the GoM [63].

The taxonomic profile revealed that phylotypes belonging to the order Nitrosopumilales predominated at all the stations, with ammonia-oxidizing archaea increasing in abundance within the ROMZ layer. This observation aligns with the previously reported abundance in low oxygen concentrations in oligotrophic ecosystems [18,61,64]. During the summer season, the epipelagic zone of the GoM is predominantly characterized by a microbial loop where the primary nitrogen source is ammonium [65] mainly consumed by high-light *Prochlorococcus* [36]. These results suggest a vertical succession from ammonia-oxidizing bacteria to ammonia-oxidizing archaea from the upper layers to the ROMZ, where *Prochlorococcus* appears in lower proportions. This aligns with the hypothesis that some *Prochlorococcus* populations may shift their function in the ROMZ, potentially utilizing nitrate, as happens in the low-light ecotype *Prochlorococcus* MIT9313 [66], the same ecotype found in our data. Furthermore, the vertical displacements of the isopycnals at the stations under extreme mesoscale structures, such as PO1-X5, an anticyclonic eddy detached from LC, and those inside the LC, such as A10-X5, Y3-X6, B18-X5, exhibited distinct taxonomic profiles. Particularly, they showed an increased relative abundance of Alteromonadales in the ROMZ. However, this increase of Alteromonadales in the mesopelagic zone (ROMZ, 800 m and 1000 m) seems to be more strongly associated with seasonality than the mesoscale structures. Their abundance tended to increase in XIXIMI-5 (early summer) and XIXIMI-6 (summer) and decrease drastically in XIXIMI-7 (late spring), and the PERMANOVA results confirmed highly significant differences between summer and late spring. Moreover, Alteromonadales have been previously reported to be affected by temporal variability [67].

### 4.2. The Large Heterogeneity in the Bathypelagic Zone Is Mainly Explained by Hydrocarbon Degraders

The bathypelagic zone of the GoM presents extreme conditions in which microbial life must thrive to survive and grow, including complete darkness, low temperatures, high hydrostatic pressure, and limited organic material input, which relies on sinking particles from the surface and metabolic byproducts of autotrophic microorganisms. The lack of easily available C sources is a limiting factor for microbial life in such an environment. Additionally, it is known that the large and heterogenous presence of natural oil effluxes and cold seeps (e.g., methane) creates a low diversity hotspot of metabolically specialized microbial groups that are able to grow using hydrocarbon-like or more recalcitrant compounds.

Based on the clustering and ordination analyses, our results showed that the prokaryotic community in the bathypelagic was more heterogenous compared to that in the mesopelagic zone. It also presented the lowest richness and diversity, and the most uneven distribution of phylotypes based on the Gini index. This heterogeneity could be attributed to the formation of micro-niches promoted by irregular spatial events such as natural oil and gas seeps. For instance, stations such as C22-X5, A4-X5, B12-X5 and C23-X7 had a high relative abundance of the gammaproteobacterium *Alcanivorax* (ranging between ~2 and 7.1% relative abundance per sample), whereas its overall abundance in the rest of the GoM was <2% (per sample basis) or absent in various cases. *Alcanivorax* (order Oceanospirillales) is an obligate hydrocarbonoclastic bacterium that was identified as one of the first groups to dominate the community during the DWH oil spill [24,25,26]. The importance of *Alcanivorax* is presumed to be due to its unique metabolic capacity for the removal of hydrocarbons from natural (e.g., oil seeps and hydrocarbon emanations) and anthropogenic sources (e.g., oil spills); some of their members are ubiquitous in marine ecosystems with specialized metabolism of a broad alkane compounds, e.g., *Alcanivorax borkumensis*, which is an obligate marine hydrocarbon-degrading bacteria that can use the C_6_ to C_18_ alkanes as a sole C source [68,69]. The enrichment of this group in specific marine stations might therefore be related to micro-niches promoted by irregular spatial events in the deep region of the GoM (see [9]).

Similarly, the gammaproteobacterium *Pseudoalteromonas* (order Alteromonadales) was present in higher abundance at some stations, such as F37-X6 y D30-X6. Its relative abundance ranged between 5% and <11% at those specific stations. *Pseudoalteromonas* was also identified as part of the hydrocarbon degradation community, i.e., a hydrocarbonoclastic bacterium. However, their enrichment followed a reduction in petroleum hydrocarbons followed by an increase in aromatic compounds [25]. This could be indicative of a possible light oil leak near these marine stations. However, some members of *Pseudoalteromonas* are also known for their copiotrophic metabolism and particle-associated nature and thus are a ubiquitous presence in marine ecosystems. So, their enrichment is associated with the presence or release of a more easily available and polar hydrocarbon-like compounds that succeed the heavier hydrocarbon compounds’ metabolism carried out for other members of the community, e.g., *Alcalinivorax*.

Other groups, also associated with hydrocarbon degradation, included the gammaproteobacterium *Acinetobacter* (order Pseudomonadales). Their phylotypes exhibited high abundance at stations such as A6-X7 and A7-X7 and matched with the increased relative abundance of *Alcanivorax* in sample C23-X7. This genus was identified as one of the predominant genera detected in the oil plume during DWH [25,70] and as a keystone hydrocarbon-degrading group in the regions of both the Perdido Fold Belt and Campeche Knolls of the GoM [27]. *Acinetobacter* encodes the alkane-hydroxylating systems (cytochrome P450 family) for the metabolism of alkanes, allowing them to grow, for instance, with extra-light oil as a solely C source as observed by [71] in reactors inoculated with seawater from the northern GoM.

Another example of this speckled environment is the presence of the genus *Erythrobacter* (order Sphingomonadales). *Erythrobacter* dominated (with ~25% relative abundance) at station D27-X6. However, reports regarding its hydrocarbon degradation capabilities are fewer compared to the aforementioned groups. Still, it is known that although it is typically present in more superficial waters, it also has a wide range of polycyclic aromatic hydrocarbon degradation capabilities in hydrothermal ecosystems [72].

In addition to hydrocarbon seepage events, the heterogeneity of bacterial and archaeal communities at the BTM may also be related to the patchy seeding of phylotypes from surface waters into the bathypelagic region via (fast) sinking particulate organic matter [73,74]. Such may be the case at stations F37-X6 (in both the 1000 m and BTM layers) and PO1-X5, which interestingly showed a high relative abundance of *Prochlorococcus* MIT9313. This group is an autotrophic cyanobacterium dominating the euphotic zone of the GoM [36]. However, another cyanobacteria (i.e., *Synechococcus* spp.) has been found at high depths in the bathypelagic layer of the Pacific Ocean, trapped in sinking particles of 0.2–3 μm particulate organic matter [75]. Interestingly, in the case of station PO1-X5, the presence of this group could also be due to the nepheloid layer previously reported at this station [76] or, in the case of F37-X6, the proximity of the Yucatan platform, which can generate rapid washouts from the platform. Sediment resuspension in this region might be associated with deep currents causing the movement and the identification of this group in the water column near the bottom of the GoM [76].

### 4.3. Unknown Phylotypes of Nitrosopumilales Dominated the Archaeal Community in the Deep Waters of the GoM

Marine archaea play a crucial role in the Earth’s biogeochemical cycles [29,30], with a significant role in the C and N cycles. However, there is a lack of information about their contribution in the deep waters of marine environments such as those in the GoM. In the Mexican EEZ, this information is limited to the waters of the Yucatan platform (180 m depth) [35] or to the sediments [77].

Our results revealed an archaeal community largely dominated by Thermoproteota (formerly Crenarchaeota), Thermoplasmatota, and Nanoarchaea. Specifically, members of Nitrosopumilales (solely unknown phylotypes of Nitrosopumilaceae; Figure 5), unknown phylotypes of the orders MG-II (currently *Candidatus* Poseidoniales) and MG-II (both from Thermoplasmatota), and members of Woesearchaeales (Nanoarchaea). These groups comprised almost the entire archaeal community across the aphotic waters in the GoM.

Thermoproteota have been widely identified in the open ocean and in the GoM [15,31,78,79], and their dominance in marine environments may be attributed to their heterotrophic metabolism, which aids in the remineralization of high-molecular-weight organic matter [80]. The dominance in the aphotic waters is a hint at their key role in the C and N pathways and mixotrophic lifestyle. One of their members, identified as one of the dominant orders in our study, i.e., Nitrosopumilales, also includes species with autotrophic and mixotrophic metabolisms that play a crucial role in the ocean biogeochemical cycles. These include the ammonia-oxidizing archaea, which are responsible for the oxidation of ammonia (NH_3_) to nitrite (NO_2_^–^), an essential step in nitrification [81].

Nitrosopumilales were abundant in all depths but with a greater presence in the mesopelagic zone. The larger abundance might help to partially explain the consumption of dissolved oxygen and the addition of nitrate by remineralization previously reported between 248 and 805 m depth in the GoM [4], in addition to the possibility that different phylotypes may respond to different oxygen concentrations with varying metabolic adaptations, as previously mentioned [67,82]. Despite the abundant presence across depths in the GoM, all the members of the Nitrosopumilaceae family remained unclassified up to the genus rank, reinforcing the limited knowledge of this group and the large proportion of uncultured members of archaea [83].

Marine groups (i.e., MG-II and MG-III, both within Thermoplasmatota), which were the second and third more abundant orders across water depths in our study, are known to participate in organic matter remineralization in the open ocean [84,85,86,87]. Recently, in silico analysis allowed the identification of a metabolic and geographical partitioning of members of the largely unknown MG-II [87]. Given the variable nutrient type across the water column in the GoM and the wide metabolism that MG-II presents, this might ensure their growth irrespective of the variable conditions at different depths.

The decremental abundance of members within Nitrosopumilales and MG-II was observed with the depth in the GoM. By contrast, MG-III, Woesearchaeales and Methanofastidiosales increased their abundance with the depth. This variability in the abundance of groups that dominated the overall archaeal community might partially explain the differences in the bathypelagic and mesopelagic zones, as observed in the ordination analyses. It also suggests that more active metabolic activity related to N metabolism might be true in more surficial waters (i.e., mesopelagic zone) compared to that of the bathypelagic zone, in which groups related to the methane cycle were more abundant. The identification of different unique phylotypes found at different discrete depths could reinforce the idea that a certain level of ecological or metabolic and functional specialization (microbial niches) across the water column might exist. These results are also in line with the observation obtained through functional prediction.

Other phylotypes ubiquitously identified through the depths but with larger abundance at the bathypelagic zone were members of the Marine Benthic Group A (MBG-A) (within Thermoproteota). The MBG-A had previously been associated solely with marine sediments and hydrothermal vents [88,89,90,91], and it was similarly identified throughout the water column in the South China Sea by [92]. By contrast, a sporadic identified case was that with members of the Geothermarchaeaceae family, which showed higher abundance at greater depths at station near the Yucatan Channel. This group has been associated with hydrothermal and geothermal environments [93,94]. However, in this study, we cannot associate its presence with hydrothermal activity in that area.

Groups associated with sediments and/or hydrothermal activity were identified at 1000 m and the BTM (Figure 5). These included methanogens (e.g., Methanofastidiosales [95,96], *Methanoregula* [97], and *Methanospirilum* [98]) and halophiles such as unknown members of the Halomicrobiaceae family [99]. The presence of these archaeal groups at greater depths in the GoM might be related to the existence of hydrothermal vents, cold seeps or marine or saline sediments in the GoM. However, the possibility that some occurrences result from inactivated or dead cell debris sinking from the surface to greater depths cannot be ruled out [73].

## 5. Conclusions

In this study, the bacterial and archaeal communities in the deep-water region of the southern GoM were assessed. The composition of the prokaryotic community was notably influenced by the AOU in the mesopelagic zone, showing significant differences in its composition and assembly in the ROMZ between the Loop Current and the south regions. The mesopelagic zone was also dominated by phylotypes of the ammonia-oxidizing archaeal group Nitrosopumilales, followed by Alteromonadales, which tended to increase in summer. In addition, communities from the bathypelagic zone appeared to be influenced by events like hydrocarbon and gas seeps and the dispersal of particulate organic matter, leading to significant heterogeneity. Archaea such as Nitrosopumilales and Methanofastidiosales likely play crucial roles in the nitrogen and carbon biogeochemical cycles of the deep GoM. Although metabarcoding analysis only provides a taxonomic background, this study is particularly significant as it marks the first report of archaea in the Mexican EEZ of the GoM and deep waters, underscoring the region’s ecological complexity. Furthermore, we offer novel insights into the role of deep-sea microorganisms in the biogeochemical processes occurring within the GoM and their potential resilience against environmental perturbations, such as natural oil seeps. However, the future integration of different omics approaches must be considered for an insightful understanding of the metabolic and functional characterization of marine microbial communities.

## Figures and Tables

**Table 1:** Seawater physicochemical characterization results.

Seawater Depth	Conservative Temperature	Absolute Salinity	Dissolved Oxygen	AOU ^1^	NO^3−^ + NO^2−^	PO_4_^3−^
	–––(°C)–––	––(g kg^−1^)––	–––––––––––––––––––(µmol kg^−1^)–––––––––––––––––––			
ROMZ (n = 32)	10.1 ^2^ ± 0.7 ^3^ a ^4^	35.41 ± 0.10 a	107 ± 5 d	166.8 ± 4 a	26.5 ± 1.7 b	1.6 ± 0.1 b
800 m (n = 15)	6.1 ± 0.5 b	35.09 ± 0.01 b	139 ± 10 c	163.8 ± 6 a	28.5 ± 1.5 a	1.8 ± 0.1 a
1000 m (n = 32)	5.1 ± 0.4 c	35.10 ± 0.01 b	166 ± 12 b	145 ± 9 b	25.6 ± 3.5 b	1.7 ± 0.1 a
BTM (n = 16)	4.1 ± 0.1 d	35.15 ± 0.00 c	203 ± 4 a	116.8 ± 4 c	22.4 ± 0.3 c	1.4 ± 0.1 c
F-value	84.68	82.41	84.46	76.05	52.40	51.34
*p*-value	<0.001	<0.001	<0.001	<0.001	<0.001	<0.001

^1^ Apparent oxygen utilization; ^2^ average value for each categorial depth; ^3^ values indicate standard deviation; ^4^ same letter indicates no significant differences between depth, i.e., within columns (Dunn test with Bonferroni–Hochberg correction *p* < 0.05). Seawater depth: ROMZ, relative oxygen minimal zone (350–600 m); 800 (800 m); 1000 (1000 m); BTM (1100–3700 m).

**Table 2:** PERMANOVA results of the beta-diversity analyses (based on the UniFrac distances) of the bacterial and archaeal communities in the deep waters of the southern Gulf of Mexico.

		Weighted UniFrac			Unweighted UniFrac		
		–––––––––––––––––––––––––––			–––––––––––––––––––––––––––		
		PERMANOVA ^1^		Betadisper ^2^	PERMANOVA		Betadisper
		–––––––––––––		–––––––––––	–––––––––––––		–––––––––––
Factor	Df ^3^	R^2^	*p*-Value	*p*-Value	R^2^	*p*-Value	*p*-Value
Depth ^4^	3	13.5	**<0.001**	0.057	17.9	**<0.001**	0.052
Campaign ^5^	2	12.7	<0.001	**0.041**	9.2	<0.001	**0.012**
Region ^6^	3	9.6	**0.003**	0.063	5.5	**<0.001**	0.112

^1^ PERMANOVA test: R^2^ and *p*-values were determined with the “adonis2” function from the vegan package based on 9999 permutations [54] (Oksanen et al. 2023). ^2^ Dispersion homogeneity analyses results (permutest and betadisper), which indicate the homogeneity of the dispersion between treatments. ^3^ Degrees of freedom. ^4^ Water column depth: ROMZ, relative oxygen minimal zone (350–600 m), 800 m, 1000 m and BTM (1100 m–3700 m). ^5^ Oceanographic campaigns: XIXIMI-05 to -07. ^6^ Regional discrete categorization of samples into “north”, “center”, “south” and “LC”. Significant *p*-values are in bold text for both PERMANOVA and betadisper results.

## Data Availability

Raw sequences are publicly available in the Sequence Read Archive (SRA) database under the NCBI’s BioProject accession number PRJNA1141103.

## Supplementary Materials

The following supporting information can be downloaded at https://www.mdpi.com/article/10.3390/microorganisms13051106/s1, Figure S1: (a) Rarefaction plot of samples (n = 95) per oceanographic campaign; and (b) scatter plot of Gini index per depth. Annotation shows the proportion of points above and below 0.5 for the Gini index; Figure S2: Beta-diversity analyses of the prokaryotic community structure. Non-metric multidimensional scaling (NMDS) of the effect of sampling region based on the (a) weighted and (b) unweighted UniFrac distances, and the effect of temporal variability (i.e., oceanographic campaigns) based on the (c) weighted and (d) unweighted UniFrac distances of the prokaryotic community. Both the R^2^ and *p*-values were determined with the “adonis2” function of the vegan package v2.6-4 [54], with 9999 permutations; Figure S3: Heatmap with the z-score transformed of the centered-log-ratio (clr) abundance of the differentially abundant ASVs between 1000 m and the BTM. Rows represent differentially abundant ASVs with a large effect size (>1), and columns represent samples. The “complete” agglomeration method based on the Euclidian distances was used for the hierarchical clustering for both samples and ASVs. Heatmap annotation (right sidebar colors) shows the effect size noted by a purple color scale, whereas columns (samples) are categorized by the 1000 m and BTM depths. Taxonomic names of ASVs indicate the nearer upper rank at which that ASV was annotated, with “c__”, “o__” and “f__” depicting the class, order and family rank, respectively; Figure S4: Archaeal community structure among the depths in the Gulf of Mexico. Bar plots displaying the relative abundance of archaeal groups collapsed at the (a) phylum and (b) order taxonomic ranks; (c) alpha diversity of the archaeal community structure: species richness (“Observed”, here ASVs), Shannon and Simpson indices; (d) intersection (upset) plot showing the number of unique (single black-filled points below the bar plots) and shared (single black-filled and connected black dots below the bar plots) ASVs among the depths (description of depth categories can be found in Figure 3); and (e) non-metric multidimensional scaling (NMDS) plots based on the weighted and unweighted UniFrac distances. The R^2^ and *p*-values were determined with the “adonis2” function of the vegan package v2.6 [54], with 999 permutations; Figure S5: Volcano plot based on the differential abundance analyses of archaeal and bacterial phylotypes. Plots correspond to the pairwise comparison between depths, with (a) ROMZ–800 m, (b) ROMZ–1000 m, (c) ROMZ–BTM, (d) 800 m–1000 m, (e) 800 m–1000 m, and (f) 1000 m–BTM. The effect size is shown on the x-axis and the expected value of the Benjamini–Hochberg corrected *p*-value on the y-axis. A negative effect size value means that the relative abundance of a metabolic pathway was larger at a shallower than a deeper depth, while a positive value indicates the opposite. The expected value of the Benjamini–Hochberg corrected *p*-value and the effect size was calculated with the “aldex.ttest” function of the ALDEx2 package v1.38 [58,60]; Table S1: Seawater samples used by stations, regions, oceanographic campaigns, and depth layers; Table S2: Marine stations, depth categorization and seawater physical characteristics; Table S3. Pairwise comparison results for the effect of the depth and sampling region on the assembly of the structure of prokaryotic community in waters of the Gulf of Mexico; Table S4. Environmental variables’ effect on the overall community; Table S5. Differential abundance analysis results of the functional prediction.

## Abbreviations

The following abbreviations are used in this manuscript:

GoM	Gulf of Mexico
EEZ	Exclusive Economic Zone
AOU	Apparent Oxygen Utilization
ROMZ	Relative Oxygen Minimum Zone
NADW	North Atlantic Deep Water
AAIW	Antarctic Intermediate Water
SUW	Subtropical Underwater
TACW	Tropical Atlantic Central Water
POM	Particulate Organic Matter
NPP	Net Primary Production
PSU	Practical Salinity Units
ASV	Amplicon Sequence Variant
NMDS	Non-Metric Multidimensional Scaling
PERMANOVA	Permutational Multivariate Analysis of Variance
VIF	Variance Inflation Factor
CTD	Conductivity–Temperature–Depth
