# Supplementary material for: The Meso- and Bathypelagic Archaeal and Bacterial Communities of the Southern Gulf of Mexico Are Dominated by Nitrifiers and Hydrocarbon Degraders
Source: Microorganisms. 2025 May 11;13(5):1106. doi: 10.3390/microorganisms13051106 (PMC12113859; doi:10.3390/microorganisms13051106)
Supplement: Supplementary file 1 [file microorganisms-13-01106-s001.zip › Table S1.pdf]

Table S1. Seawater samples used by stations, regions, oceanographic campaigns, and depth layers

		**Oceanographic campaigns**											
Station	Region	XIXIMI-05				XIXIMI-06				XIXIMI-07			
		ROMZ	800m	1,000 m	BTM	ROMZ	800m	1,000 m	BTM	ROMZ	800m	1,000 m	BTM
A2	North									×		×	
A4	North	×		×	×								
A6	North									×		×	×
A7	North	×		×	×					×		×	×
A10	North	×		×	×			×	×		×	×	
B12	North	×	×	×						×	×	×	
B17	North	×	×	×		×	×						
B18	North	×	×	×									
C21	Center					×			×				
C22	Center	×		×	×								
C23	Center									×		×	×
C24	Center									×	×	×	
C25	Center					×	×	×					
D27	Center					×		×	×				
D30	Center					×		×	×				
E33	Center					×	×	×					
F37	South					×		×	×				
G40	South					×	×	×					
G44	South	×	×	×		×	×	×					
H45	South					×	×	×					
H46	South	×		×	×								
H47	South	×	×	×									
P01	LC	×		×	×								
TS1	North	×	×	×									
Y3	LC					×							
Y6	LC									×		×	×
Y7	LC					×		×	×	×		×	
Y9	LC											×	

Collection depths: ROMZ, relative oxygen minimal zone (350-600 m); 800 m; 1,000 m; and BTM, bottom waters (1,100 – 3,700 m). LC: Loop Current.
