# Supplementary material for: The Meso- and Bathypelagic Archaeal and Bacterial Communities of the Southern Gulf of Mexico Are Dominated by Nitrifiers and Hydrocarbon Degraders
Source: Microorganisms. 2025 May 11;13(5):1106. doi: 10.3390/microorganisms13051106 (PMC12113859; doi:10.3390/microorganisms13051106)
Supplement: Supplementary file 1 [file microorganisms-13-01106-s001.zip › Table S2.pdf]

Table S2. Marine stations, depth categorization and seawater physical characteristics

Station	Depth layer [1]	Oceanographic campaign	Sampling depth (m)	Seafloor depth (m)	Density anomaly (kg m^{-3})
A10	ROMZ	XIXIMI-05	510.4	3362	27.09
A4	ROMZ	XIXIMI-05	453.5	3629	27.11
A7	ROMZ	XIXIMI-05	452.7	3525	27.09
B12	ROMZ	XIXIMI-05	449.7	3564	27.07
B17	ROMZ	XIXIMI-05	500.1	3088	27.19
B18	ROMZ	XIXIMI-05	414.6	1275	27.17
C22	ROMZ	XIXIMI-05	474.4	3728	27.11
G44	ROMZ	XIXIMI-05	421.7	2387	27.10
H46	ROMZ	XIXIMI-05	358.3	2751	27.14
H47	ROMZ	XIXIMI-05	415.1	1489	27.07
PO1	ROMZ	XIXIMI-05	633.3	3287	27.01
TS1	ROMZ	XIXIMI-05	383.7	1860	27.10
B12	800 m	XIXIMI-05	800.5	3564	27.39
B17	800 m	XIXIMI-05	799.6	3088	27.47
B18	800 m	XIXIMI-05	799.4	1261	27.57
G44	800 m	XIXIMI-05	802.5	2387	27.46
H47	800 m	XIXIMI-05	799.7	1489	27.48
TS1	800 m	XIXIMI-05	798.8	1860	27.52
A10	1,000 m	XIXIMI-05	999.4	3362	27.61
A4	1,000 m	XIXIMI-05	1000.6	3629	27.61
A7	1,000 m	XIXIMI-05	1000.6	3525	27.60
B12	1,000 m	XIXIMI-05	1002.7	3564	27.56
B17	1,000 m	XIXIMI-05	1004.6	3088	27.60
B18	1,000 m	XIXIMI-05	1002.6	1275	27.68
C22	1,000 m	XIXIMI-05	999.6	3728	27.58
G44	1,000 m	XIXIMI-05	999.6	2387	27.60

H46	1,000 m	XIXIMI-05	998.1	2751	27.63
H47	1,000 m	XIXIMI-05	1000	1489	27.61
PO1	1,000 m	XIXIMI-05	1000.4	3287	27.47
TS1	1,000 m	XIXIMI-05	1002.9	1860	27.63
A10	BTM	XIXIMI-05	3340.4	3362	27.72
A4	BTM	XIXIMI-05	3602.1	3629	27.72
A7	BTM	XIXIMI-05	3513.6	3525	27.72
C22	BTM	XIXIMI-05	3663.8	3728	27.72
H46	BTM	XIXIMI-05	2721.3	2751	27.73
PO1	BTM	XIXIMI-05	3276.2	3287	27.73
B17	ROMZ	XIXIMI-06	389.5	2964	27.09
C21	ROMZ	XIXIMI-06	420.8	3201	27.09
C25	ROMZ	XIXIMI-06	430.8	3721	27.14
D27	ROMZ	XIXIMI-06	414.7	2710	27.14
D30	ROMZ	XIXIMI-06	419.0	3316	27.12
E33	ROMZ	XIXIMI-06	427.4	3435	27.13
F37	ROMZ	XIXIMI-06	399.3	3186	27.10
G40	ROMZ	XIXIMI-06	449.8	1928	27.10
G44	ROMZ	XIXIMI-06	414.5	2408	27.12
H45	ROMZ	XIXIMI-06	409.4	2187	27.12
Y3	ROMZ	XIXIMI-06	513.3	1114	27.16
Y7	ROMZ	XIXIMI-06	600.4	1914	27.14
B17	800 m	XIXIMI-06	799.3	2964	27.48
C25	800 m	XIXIMI-06	800.5	3721	27.49
E33	800 m	XIXIMI-06	799.2	3435	27.49
G40	800 m	XIXIMI-06	802.2	1928	27.41
G44	800 m	XIXIMI-06	800.1	2408	27.46
H45	800 m	XIXIMI-06	800.2	2187	27.51
A10	1,000 m	XIXIMI-06	954.7	3348	27.41
C25	1,000 m	XIXIMI-06	1000.5	3721	27.61
D27	1,000 m	XIXIMI-06	999.2	2710	27.65
D30	1,000 m	XIXIMI-06	1000.1	3316	27.60

E33	1,000 m	XIXIMI-06	1000.7	3435	27.63
F37	1,000 m	XIXIMI-06	1000.1	3186	27.62
G40	1,000 m	XIXIMI-06	993.7	1928	27.59
G44	1,000 m	XIXIMI-06	1001.1	2408	27.59
H45	1,000 m	XIXIMI-06	999.5	2187	27.63
Y7	1,000 m	XIXIMI-06	1000.9	1914	27.61
A10	BTM	XIXIMI-06	3342.3	3348	27.73
C21	BTM	XIXIMI-06	3185.5	3201	27.73
D27	BTM	XIXIMI-06	2701	2710	27.73
D30	BTM	XIXIMI-06	3316.4	3316	27.73
F37	BTM	XIXIMI-06	3164.8	3186	27.73
Y7	BTM	XIXIMI-06	1892.7	1914	27.74
A2	ROMZ	XIXIMI-07	437.8	3616	27.07
A6	ROMZ	XIXIMI-07	398.2	3528	27.08
A7	ROMZ	XIXIMI-07	347.9	3527	27.04
B12	ROMZ	XIXIMI-07	423.1	3539	26.99
C23	ROMZ	XIXIMI-07	417.8	3739	27.05
C24	ROMZ	XIXIMI-07	402.2	3736	27.06
Y6	ROMZ	XIXIMI-07	526.1	1800	27.14
Y7	ROMZ	XIXIMI-07	587.9	1916	27.17
A10	800 m	XIXIMI-07	797.1	3342	27.30
B12	800 m	XIXIMI-07	794.2	3539	27.42
C24	800 m	XIXIMI-07	798.4	3736	27.48
A10	1,000 m	XIXIMI-07	996.7	3341	27.51
A2	1,000 m	XIXIMI-07	1196.7	3616	27.67
A6	1,000 m	XIXIMI-07	996.2	3528	27.62
A7	1,000 m	XIXIMI-07	995.7	3527	27.65
B12	1,000 m	XIXIMI-07	964.5	3539	27.56
C23	1,000 m	XIXIMI-07	995.6	3739	27.59
C24	1,000 m	XIXIMI-07	994.9	3736	27.60
Y6	1,000 m	XIXIMI-07	1194.9	1800	27.70
Y7	1,000 m	XIXIMI-07	996.1	1916	27.60

Y9	1,000 m	XIXIMI-07	995.4	4481	27.46
A6	BTM	XIXIMI-07	3478.7	3528	27.73
A7	BTM	XIXIMI-07	3485.7	3527	27.73
C23	BTM	XIXIMI-07	3683.9	3739	27.72
Y6	BTM	XIXIMI-07	1786.9	1800	27.74

[1] Depth layer: ROMZ, relative oxygen minimal zone (350-600 m); 800 m; 1,000 m, and BTM (> 1,100 – 3,700 m).
