# Supplementary material for: The Meso- and Bathypelagic Archaeal and Bacterial Communities of the Southern Gulf of Mexico Are Dominated by Nitrifiers and Hydrocarbon Degraders
Source: Microorganisms. 2025 May 11;13(5):1106. doi: 10.3390/microorganisms13051106 (PMC12113859; doi:10.3390/microorganisms13051106)
Supplement: Supplementary file 1 [file microorganisms-13-01106-s001.zip › Table S3.pdf]

Table S3. Pair-wise comparison results for the effect of depth and sampling region on the assembly of the structure of prokaryotic community in waters of the Gulf of Mexico.

	Weighted UniFrac		Unweighted UniFrac	
Comparison	R^2	P-value [2]	R^2	P-value [2]
**Depth [1]**				
ROMZ vs 800 m	0.046	0.474	0.205	**0.006**
ROMZ vs 1,000 m	0.091	**0.018**	0.101	**0.006**
ROMZ vs BTM	0.180	**0.006**	0.090	**< 0.001**
800 m vs 1,000 m	0.042	0.678	0.045	0.024
800 m vs BTM	0.137	**0.042**	0.134	**0.006**
1,000 m vs BTM	0.059	0.234	0.143	**0.006**
**Sampling region**				
LC Vs South	0.143	**0.001**	0.061	**0.001**
Center Vs South	0.118	**0.002**	0.053	**0.002**
LC Vs North	0.054	0.050	0.036	**0.012**
North Vs South	0.046	0.082	0.032	0.054
LC Vs Center	0.033	0.174	0.028	0.091
North Vs Center	0.028	0.184	0.023	0.314
For each depth				
**ROMZ**	0.179	**0.048**	0.129	**0.009**
LC Vs South	0.145	**0.005**	0.138	**0.002**
Center Vs South	0.121	**0.034**	0.094	**0.024**
LC Vs North	0.053	**0.050**	0.101	**0.035**
North Vs South	0.047	0.088	0.087	0.080
LC Vs Center	0.033	0.212	0.071	0.210
North Vs Center	0.027	0.240	0.064	0.236
**800 m**	0.245	0.308	0.213	0.467
**1,000 m**	0.216	**0.016**	0.213	0.467
LC Vs South	0.285	**0.006**	0.106	**0.021**
Center Vs South	0.385	**0.003**	0.110	0.064

LC Vs North	0.037	0.588	0.083	0.088
North Vs South	0.158	0.063	0.063	0.455
LC Vs Center	0.070	0.351	0.081	0.137
North Vs Center	0.073	0.290	0.095	0.093
**BTM**	0.225	0.323	0.232	0.126

[1] Depth in the water column: ROMZ, relative oxygen minimal zone (350-600 m); 800 m; 1,000 m and BTM (> 1,100 – 37,00 m); [2] The PERMANOVA's test results for the R^2 (explained variance) and the *P*-value < 0.05. Both were determined with the "adonis2" function of the vegan package v2.6. [54] with 9,999 permutations.

References

54. Oksanen, J.; Simpson, G.L.; Blanchet, F.G.; Kindt, R.; Legendre, P.; Minchin, P.R.; O'Hara, R.B.; Solymos, P.; Stevens, M.H.H.; Szoecs, E.; et al. Vegan: Community Ecology Package 2024. Available at https://cran.r-project.org/web/packages/vegan/index.html.
