# Supplementary material for: The Meso- and Bathypelagic Archaeal and Bacterial Communities of the Southern Gulf of Mexico Are Dominated by Nitrifiers and Hydrocarbon Degraders
Source: Microorganisms. 2025 May 11;13(5):1106. doi: 10.3390/microorganisms13051106 (PMC12113859; doi:10.3390/microorganisms13051106)
Supplement: Supplementary file 1 [file microorganisms-13-01106-s001.zip › Table S4.pdf]

Table S4. Environmental variables' effect on the overall community, i.e., considering all amplicon sequence variants (ASVs), the dominant biosphere (DB), and rare biosphere (RB) based on ANOVA-like permutation test results

Environmental variable	Entire community (All ASVs)		Dominant biosphere [1]		Rare biosphere [2]	
	F-value	*P*-value	F-value	*P*-value	F-value	*P*-value
Seafloor depth	1.501	0.116	0.010	0.091	0.014	**0.030**
Depth	20.768	< **0.001**	0.236	< **0.001**	0.255	< **0.001**
Absolute salinity	24.638	< **0.001**	0.122	< **0.001**	0.145	< **0.001**
Conservative temperature	6.123	< **0.001**	0.039	< **0.003**	0.021	**0.005**
AOU	2.899	**0.006**	0.026	**0.001**	0.018	**0.011**
Latitud	1.374	0.150	0.025	**0.001**	0.004	0.610
Longitud	1.635	0.084	0.013	**0.044**	0.009	0.125
NO_3^-+NO_2^-	0.811	0.674	0.002	0.931	0.005	0.405
PO_4^{3-}	1.627	0.083	0.018	0.010	0.009	0.129

[1] Dominant biosphere (DB) defined as the ASVs with a representation of ≥1% relative abundance within a sample, and with an average relative abundance ≥ 0.1% per depth layer;

[2] Rare biosphere (RB) defined as the ASVs with a representation of ≤ 0.01% within a sample, and with an average relative abundance ≤ 0.001% per depth layer.

The F-value and P-value were determined to assess the statistical significance of the ANOVA-like models in evaluating the impact of physicochemical parameters on microbial composition variation ($P < 0.05$). Significant *p*-values are in bold text.
