# Supplementary material for: The Meso- and Bathypelagic Archaeal and Bacterial Communities of the Southern Gulf of Mexico Are Dominated by Nitrifiers and Hydrocarbon Degraders
Source: Microorganisms. 2025 May 11;13(5):1106. doi: 10.3390/microorganisms13051106 (PMC12113859; doi:10.3390/microorganisms13051106)
Supplement: Supplementary file 1 [file microorganisms-13-01106-s001.zip › Table S5.pdf]

Table S5. Differential abundance analysis results of functional prediction (metabolic pathways) as determined with PICRUSt2 [50] between the relative oxygen minimum zone (ROMZ) and deep waters (BTM) of the Gulf of Mexico.

Pathway	Effect size
Methanogenesis from H_2 And CO_2	-1.1
Sucrose degradation II (sucrose synthase)	-1.0
Coenzyme B biosynthesis	1.0
2-Nitrobenzoate degradation I	1.0
Creatinine degradation I	1.1
Adp-L-Glycero-Β-D-Manno-Heptose biosynthesis	1.1
L-Tryptophan degradation IX	1.1
Catechol degradation to 2-Oxopent-4-enoate II	1.1
Kdo transfer to lipid IVa III (Chlamydia)	1.1
Catechol degradation II (meta-cleavage pathway)	1.1
2-Aminophenol degradation	1.1
L-Tryptophan degradation XII(*Geobacillus*)	1.2
Nitrate reduction VI (assimilatory)	1.2
Superpathway of thiamin diphosphate biosynthesis II	1.3
Thiazole biosynthesis II (*Bacillus*)	1.3
L-Valine degradation I	1.3
L-Tryptophan degradation to 2-amino-3-carboxymuconate semialdehyde	1.4
S-Adenosyl-L-Methionine cycle I	1.4
NAD Biosynthesis II (from tryptophan)	1.4
Super pathway of thiamin diphosphate biosynthesis I	1.4
Thiazole biosynthesis I (*E. coli*)	1.5
Beta;-Alanine biosynthesis II	1.5
Myo-Inositol degradation I	1.5
Myo-, Chiro- And Scillo-Inositol degradation	1.7
Glycine betaine degradation I	2.0
Super pathway of sulfolactate degradation	2.3

References

50. Douglas, G.M.; Maffei, V.J.; Zaneveld, J.R.; Yurgel, S.N.; Brown, J.R.; Taylor, C.M.; Huttenhower, C.; Langille, M.G.I. PICRUSt2 for Prediction of Metagenome Functions. Nat. Biotechnol. 2020, 38, 685–688, doi:10.1038/s41587-020-0548-6.
