# Supplementary figures and images for: The Meso- and Bathypelagic Archaeal and Bacterial Communities of the Southern Gulf of Mexico Are Dominated by Nitrifiers and Hydrocarbon Degraders
Source: Microorganisms. 2025 May 11;13(5):1106. doi: 10.3390/microorganisms13051106 (PMC12113859; doi:10.3390/microorganisms13051106)

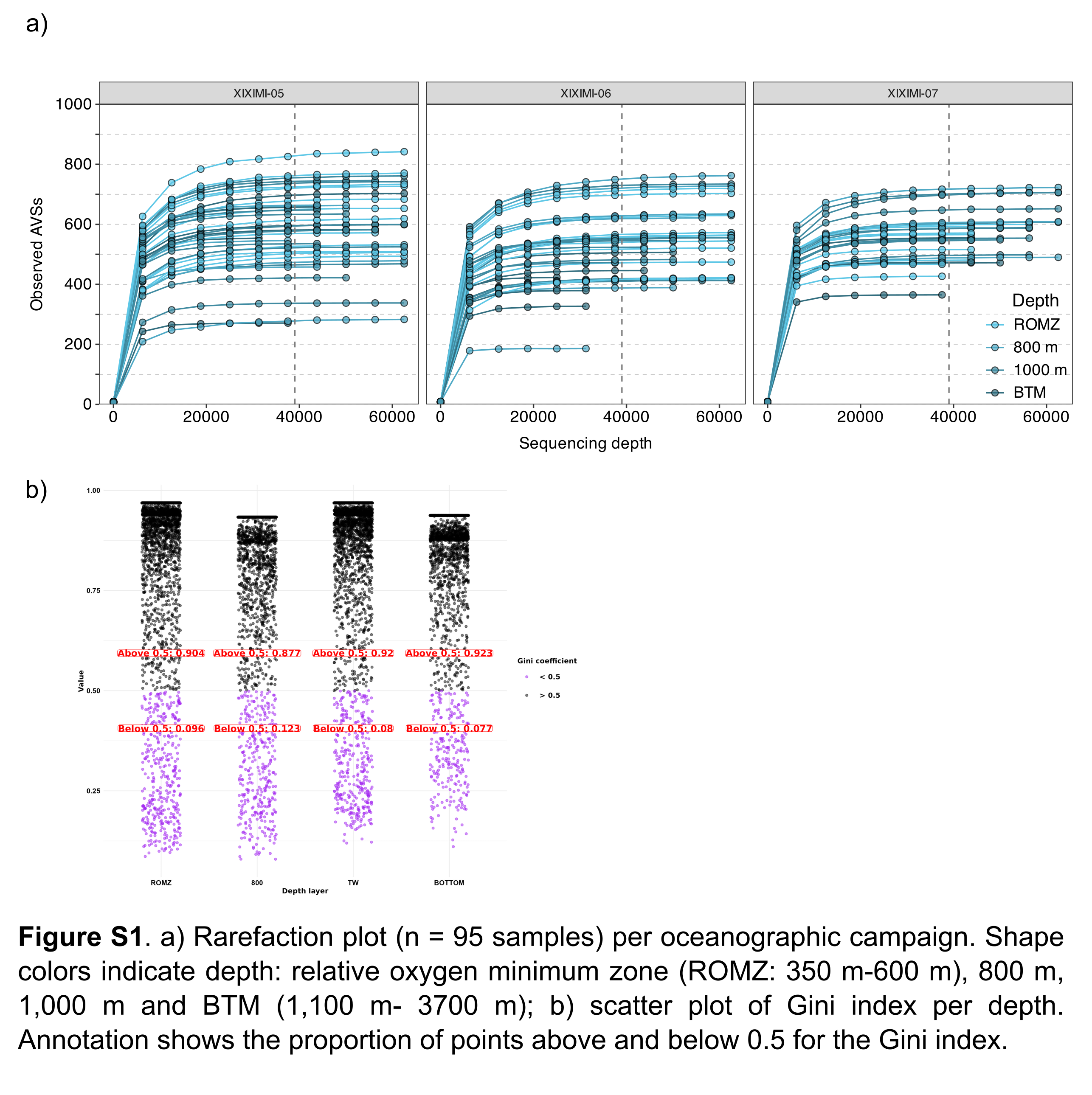

Supplement: Supplementary file 1 [file microorganisms-13-01106-s001.zip › Figure S1.png]

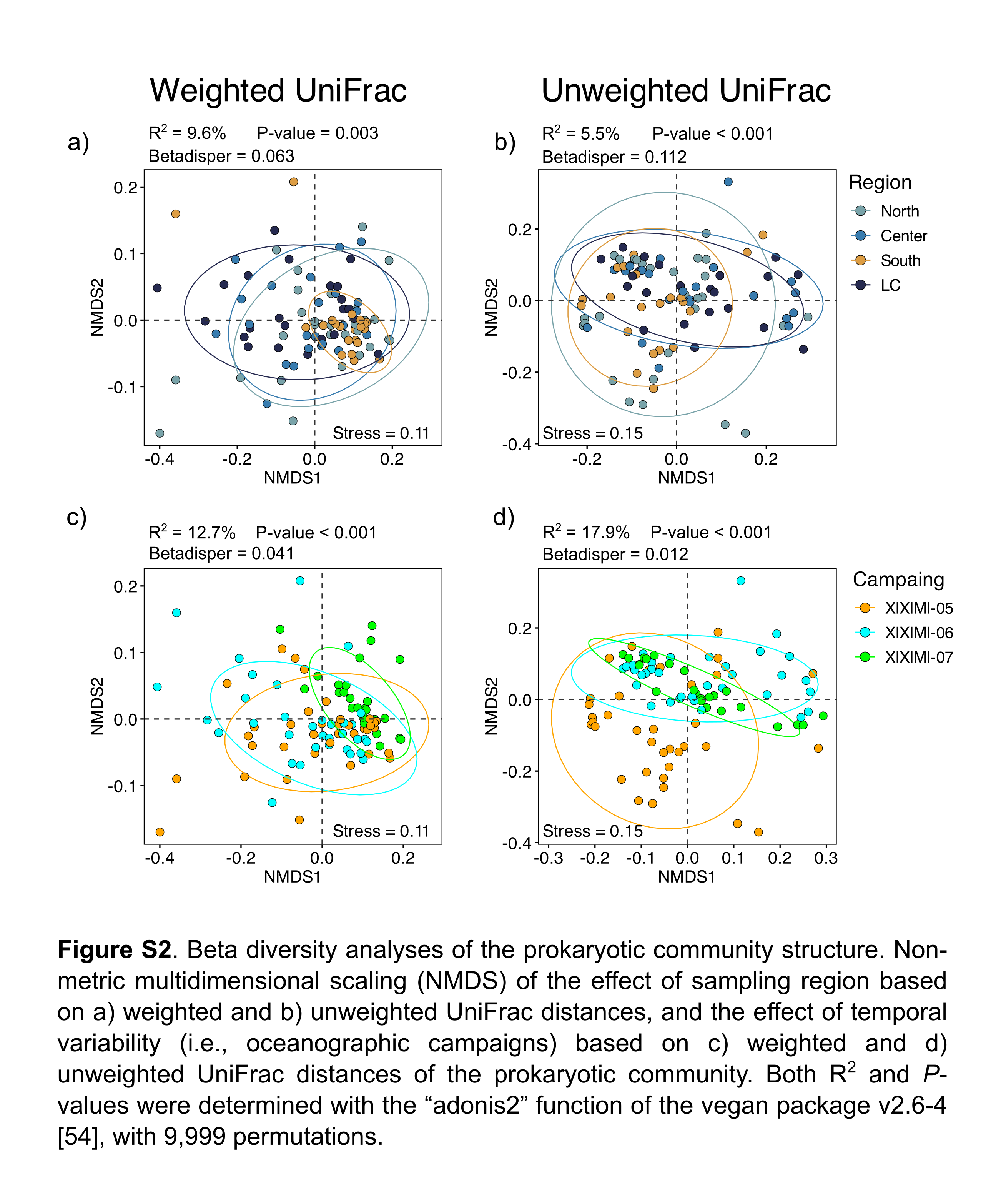

Supplement: Supplementary file 1 [file microorganisms-13-01106-s001.zip › Figure S2.png]

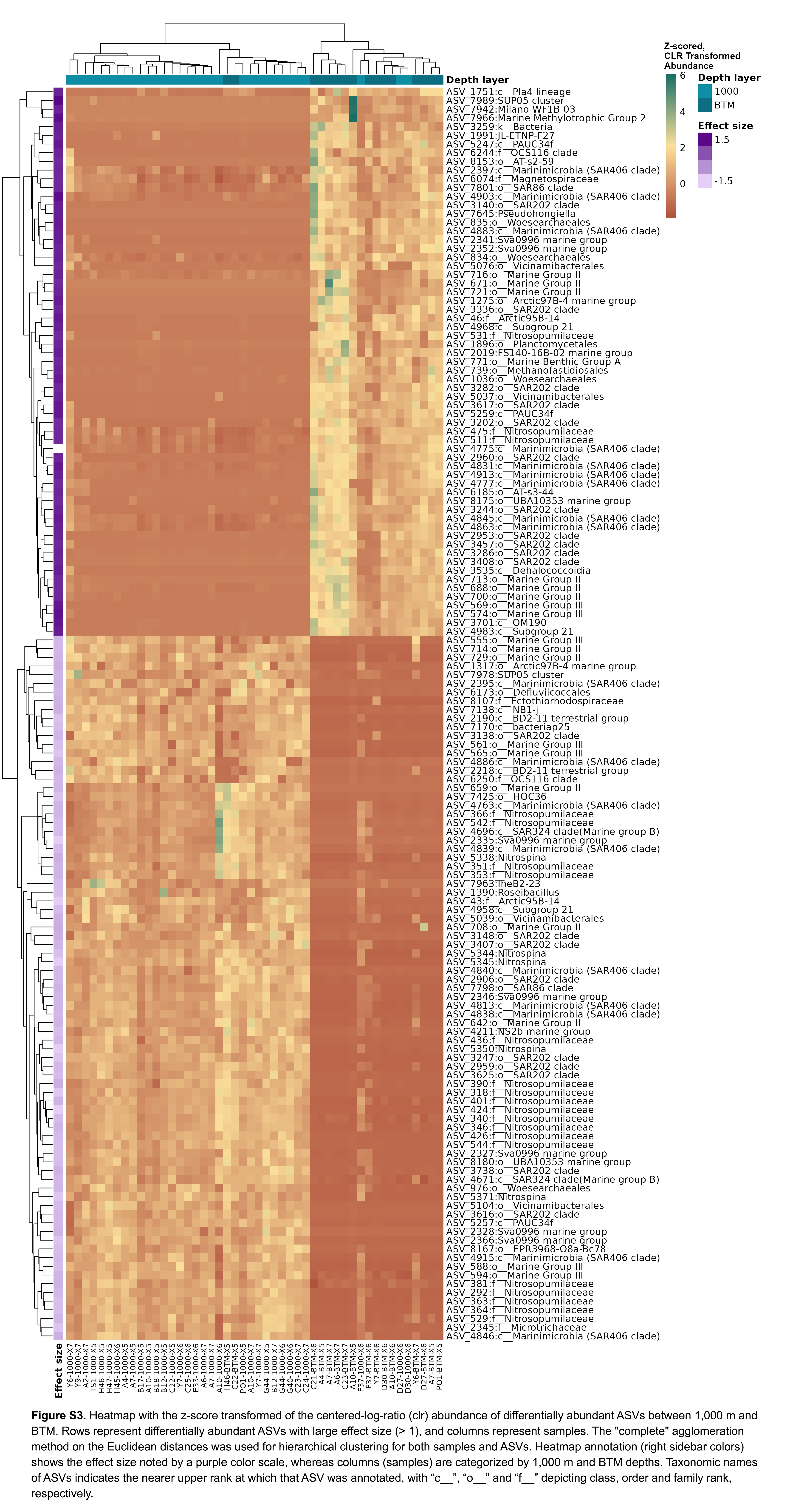

Supplement: Supplementary file 1 [file microorganisms-13-01106-s001.zip › Figure S3.png]

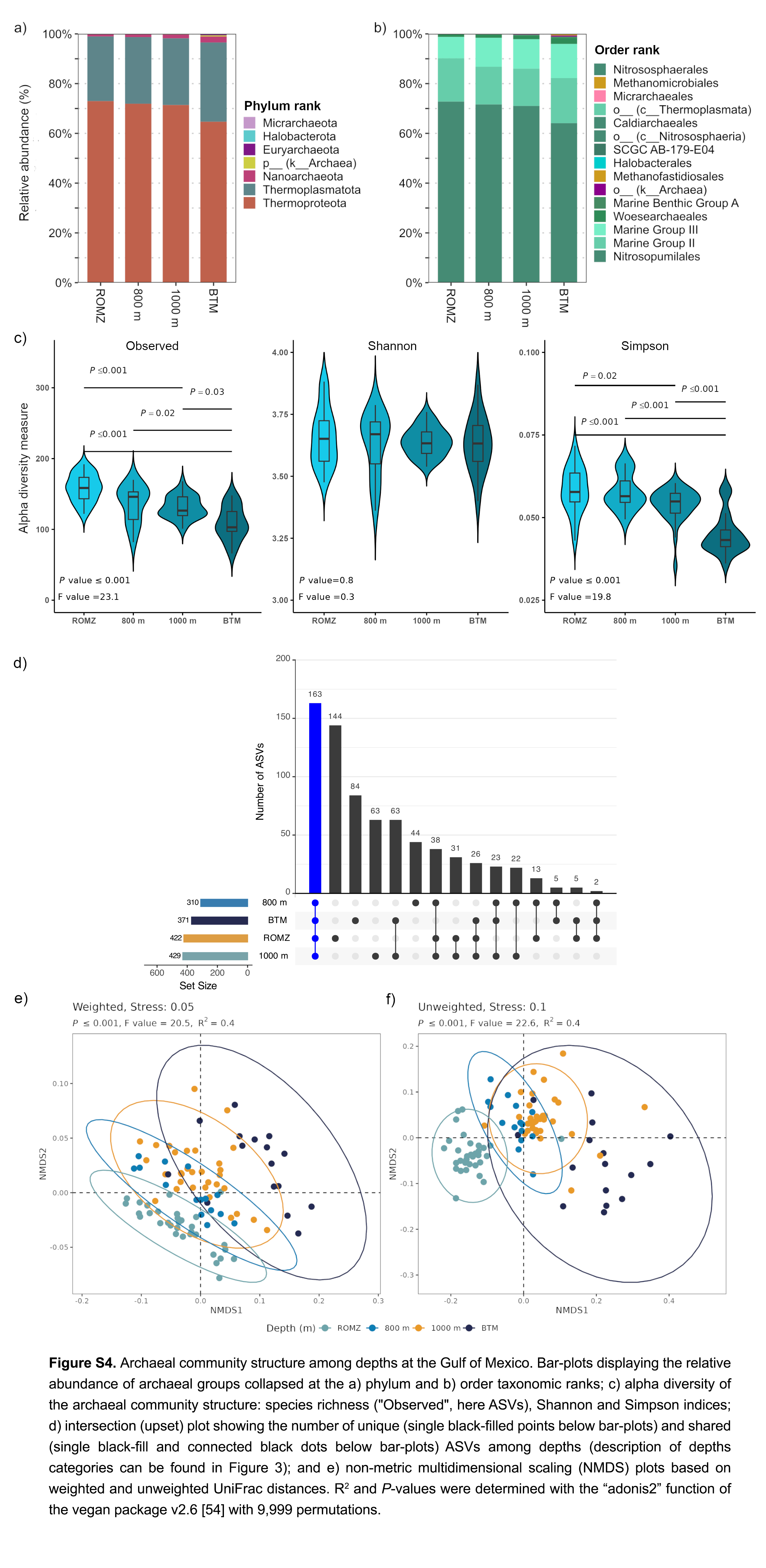

Supplement: Supplementary file 1 [file microorganisms-13-01106-s001.zip › Figure S4.png]

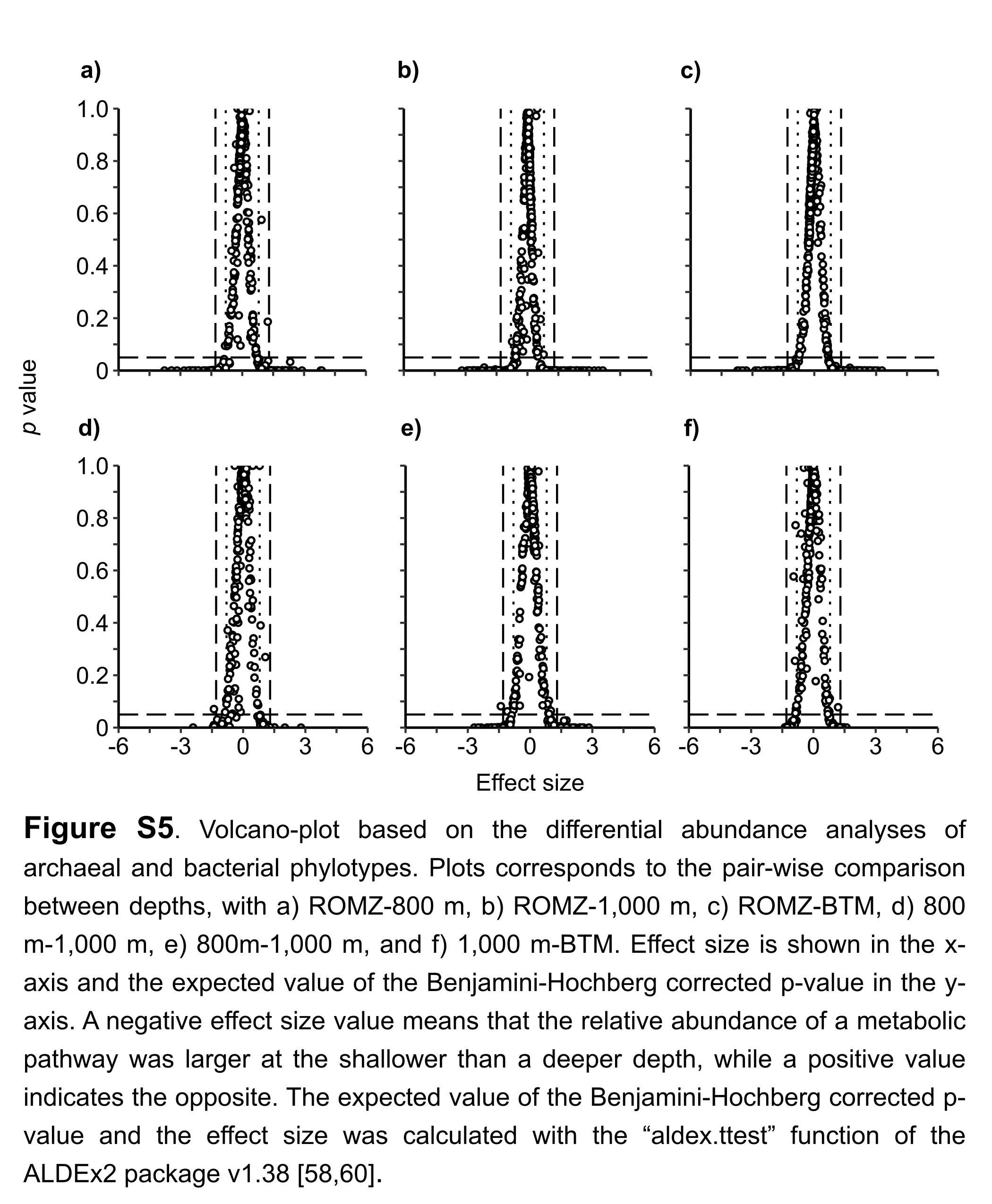

Supplement: Supplementary file 1 [file microorganisms-13-01106-s001.zip › Figure S5.png]
